# Principled Limitations on Self-Representation for Generic Physical Systems

**DOI:** 10.3390/e26030194

**Published:** 2024-02-24

**Authors:** Chris Fields, James F. Glazebrook, Michael Levin

**Affiliations:** 1 Allen Discovery Center, Tufts University, Medford, MA 02155, USA; michael.levin@tufts.edu; 2 Department of Mathematics and Computer Science, Eastern Illinois University, Charleston, IL 61920, USA; jfglazebrook@eiu.edu; 3 Adjunct Faculty, Department of Mathematics, University of Illinois at Urbana-Champaign, Urbana, IL 61801, USA

**Keywords:** free energy principle, Gödel’s theorem, Moore’s theorem, quantum reference frame, Rice’s theorem, separability

## Abstract

The ideas of self-observation and self-representation, and the concomitant idea of self-control, pervade both the cognitive and life sciences, arising in domains as diverse as immunology and robotics. Here, we ask in a very general way whether, and to what extent, these ideas make sense. Using a generic model of physical interactions, we prove a theorem and several corollaries that severely restrict applicable notions of self-observation, self-representation, and self-control. We show, in particular, that adding observational, representational, or control capabilities to a meta-level component of a system cannot, even in principle, lead to a complete meta-level representation of the system as a whole. We conclude that self-representation can at best be heuristic, and that self models cannot, in general, be empirically tested by the systems that implement them.

## 1. Introduction

To what extent can human beings be said to represent themselves? To what extent can other organisms, or more generally, other living systems, be said to represent themselves? While the definition of “representation” is philosophically controversial (Chapter 4 in Ref. [1]), here, we use this term just to mean “a description encoded as classical data”, where the relevant sense of “description” is the one employed in physics or computer science [2]. With this definition, we can ask the above questions even more generally: to what extent can a generic physical system *S* observe, represent, and control its own internal processes? To what extent can a metaprocessor $S_{1}$ observe, represent, and control the internal processes of an object-level processor $S_{2}$ with which it is associated by the architecture of some overall system *S*? These questions obviously bear on the question of the extent to which autonomous agents can be considered “self-aware” when acting. As work in basal cognition, synthetic biology, artificial intelligence (AI), and various hybrid technologies [3,4] steadily expands the scope of agentive systems, such questions increasingly have technological as well as psychological relevance. The theory of active inference driven by the Free Energy Principle (FEP) provides a fully general account of such systems within either classical [5,6,7] or quantum [8] formalisms; see Ref. [9] for a detailed comparison of the two. Hence, these questions about self-observation, representation, and control are fundamental to the theory of active inference.

We approach these questions in full generality, using a minimal-assumption representation of a finite physical system *S* that interacts with a finite environment *E*. We employ the quantum formalism outlined in Refs. [8,9]; see Refs. [10,11,12] for additional details on this formalism. This quantum formalism is more general than the classical formalism, which is obtained in the classical limit as described in Refs. [8,9]. Using this general approach, we prove four “no-go” results that severely limit the extent to which a system can be said to observe, represent, or control its own internal processes. These results can be seen as analogs of previous results that limit the extent to which a system can observe, represent, or control its environment [13]; from a historical perspective, they are in the lineage of Ashby’s Law of Requisite Variety [14] as well as the more specific results of Refs. [15,16]. Together with their corollaries, these results effectively limit systems to untestable, heuristic models of their own internal processes that cannot, even in principle, be extended to completeness. We conclude that “self knowledge” is fundamentally confabulatory, whether in humans or in any other systems.

## 2. Representation of Generic Physical Interactions

We begin by providing a description of physical interaction that makes no assumptions about the structures or properties of the interacting systems. A “system” in this description is just a collection of degrees of freedom that can have various values; a “state” of a system is an assignment of particular values to each of its degrees of freedom. Systems interact by acting on each other to change each other’s states. The idea of interaction requires the interacting systems—here we call them *S* and *E* for “system” and “environment”—to be distinguishable; hence, we can talk about a boundary—which we label B—that separates them. Quantum theory provides a way to express these intuitive ideas in a way that is precise enough to derive significant consequences. It provides, in particular, a criterion for deciding whether *S* and *E* can be considered separate systems, and therefore mutually conditionally independent, that will play a central role in the sections that follow.

Let *U* be a finite physical system, the states of which can be described as vectors in a Hilbert space $H_{U}$, and consider a bipartite decomposition U=SE, or more explicitly, $H_{U}=H_{S}⊗H_{E}$. We can then write the internal or self-interaction of *U*, represented by a Hamiltonian operator $H_{U}$, as $H_{U}=H_{S}+H_{E}+H_{SE}$, where $H_{S}$ and $H_{E}$ are the internal interactions of *S* and *E*, respectively, and $H_{SE}$ is the interaction between them. We are interested in the case in which $H_{SE}$ is weak enough that most degrees of freedom of *S*, and most degrees of freedom of *E*, are not involved directly in the interaction. In this case, both *S* and *E* have well-defined “internal states” which we will designate (using the Dirac notation) |S〉 and |E〉, respectively, with $\rho_{S}$ and $\rho_{E}$, the corresponding state densities. A necessary and sufficient condition for this case is that the joint state |U〉=|SE〉 is *separable*, i.e., factors as |SE〉=|S〉|E〉; this condition corresponds, by definition, to |SE〉 being unentangled. Entanglement or non-separability is not an “objective” or observer-independent condition of a system but rather depends on how the joint state |SE〉 of the system is described [17,18,19,20]. The availability of a description under which |S〉 and |E〉, or $\rho_{S}$ and $\rho_{E}$, are separable guarantees that they are, under that description, conditionally independent as required by the classical FEP [8].

**Figure 1:**
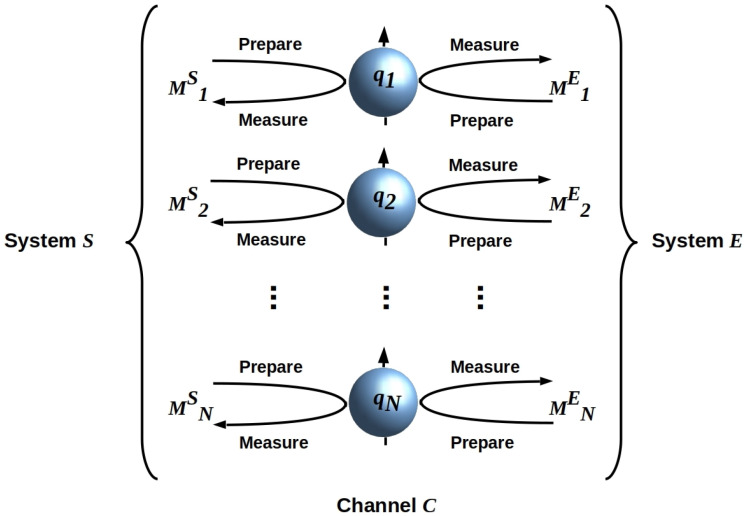
A holographic screen B separating systems *S* and *E* with an interaction $H_{SE}$ given by Equation (1) can be realized by an ancillary array of noninteracting qubits that are alternately prepared by *S* (*E*) and then measured by *E* (*S*). Qubits are depicted as Bloch spheres [25]. There is no requirement that *S* and *E* share preparation and measurement bases, i.e., quantum reference frames as discussed below. Adapted from Ref. [10], CC-BY license.

Given a description under which |SE〉 is separable, we can choose basis vectors $|i_{k}\rangle$, k=S or *E*, and write the interaction as:

(1) $$H_{SE}=N\beta_{k}k_{B}T_{k}\sum_{i}^{N}\alpha_{i}^{k}M_{i}^{k},$$

where $k_{B}$ is Boltzmann’s constant, $T_{k}$ is the temperature, the $\alpha_{i}^{k}\in \left[0,1\right]$ are such that $\sum_{i}^{N}\alpha_{i}^{k}=1$, the $M_{i}^{k}$ are *N* Hermitian operators with eigenvalues in {−1,1}, and $\beta_{k}\geq \ln2$ is an inverse measure of *k*’s thermodynamic efficiency that depends on the internal dynamics $H_{k}$. The operators $M_{i}^{k}$ can be interpreted as measurement, or dually, Ref. [21] state-preparation operators, each acting on a single quantum bit (qubit) $q_{i}$. This allows a straightforward topological interpretation of Equation (1). Let B denote the decompositional boundary between *S* and *E*, i.e., the boundary given implicitly by the Hilbert space factorization $H_{U}=H_{S}⊗H_{E}$. Given separability, i.e., |SE〉=|S〉|E〉, the entanglement entropy S(|SE〉) across B is zero. The holographic principle (HP) constrains information exchange between separable systems to the information that can be encoded on their mutual boundary [22,23,24]; see Ref. [12] for details of how the HP applies in this setting. We can, therefore, regard B as a holographic screen, i.e., an ancillary *N*-qubit array, separating *S* from *E*, and depict $H_{SE}$ as in Figure 1.

Provided *S* and *E* are separable (classically, provided they are conditionally independent), i.e., provided B functions as a holographic screen separating *S* from *E*, we can represent B by a Hilbert space $H_{B}$ and hence assign it an *N*-qubit state |B〉. The dimension $\dim\left(H_{B}\right)=2^{N}=\dim\left(H_{SE}\right)$. The separability condition can then be restated as $\dim\left(H_{B}\right)\ll \dim\left(H_{S}\right),\dim\left(H_{E}\right)$. As B is, by definition, just a decompositional boundary—an abstract mathematical construct, not a physical surface—its Hilbert space $H_{B}$ is completely ancillary to *S* and *E*, i.e., $H_{B}\cap H_{U}=\emptyset$. Thus, while B has the function of a classical Markov blanket (MB) [26,27], limiting information exchange between *S* and *E* to *N* bits, its states are not within the physical *S*-*E* state space. A classical MB is obtained in the current setting by embedding B, *S*, and *E* in a geometric “physical” (e.g., 3d) space and considering the qubits $q_{i}$ to be “transducer” or “input/output” (I/O) states causally separating *S* from *E*. If we consider each of these transducer states to be a photon state, B becomes a light sheet causally separating *S* from *E*, as in the covariant definition of a holographic screen [24]. Note that this is the classical limit of the boundary B itself, not the classical limit of any state encoded on the boundary. If *S* and *E* are separable, they are mutually decoherent by definition. From the perspective of either *S* or *E*, B encodes classical information—observational outcomes—as Equation (1) makes clear; see Ref. [12] for further discussion.

As each of the operators $M_{i}^{k}$ on B has eigenvalues +1 and −1, we can consider each of them to be an instance $\sigma_{z}^{k,i}$ of the *z*-spin operator $\sigma_{z}$. Choosing the basis $\left\{|i_{k}\rangle \right\}$ is, effectively, choosing the local *z* axis that renders $\sigma_{z}^{k,i}$ well defined. We showed in Ref. [12] that the “free choice” of basis for each of *S* and *E* is a necessary condition for separability; if the choice of basis for *S* determines the choice of basis for *E* or vice versa, the two are entangled. As the FEP requires separability, all active inference agents are “free agents” in this fundamental, physical sense; see Ref. [28] for an alternative derivation of this result.

## 3. Quantum Reference Frames and Noncommutativity

Having described interaction in terms of elementary operations of preparation and measurement defined at the boundary B separating a system *S* from its environment *E*, we now turn to the question of how meaningful information—“differences that make a difference” [29]—is extracted from this process. Meaningful measurements are always “with respect to” something, a standard of comparison or, more technically, a *reference frame* that has a pre-established significance. For example, measurements of length require a standard, such as a meter stick, that has a fixed length that gives a standardized, actionable meaning to a measurement outcome of so many meters. Such standards must be physically implemented to be useful; any implemented reference frame is a quantum system and hence, a *quantum* reference frame (QRF) [30,31]. The formalism of QRFs gives, therefore, a principled way of talking about the extraction of meaningful information from measurements. A key question about this process is whether measurements can be made simultaneously, i.e., whether the QRFs being employed commute. The noncommutativity of QRFs induces context effects that can render the interpretation of measurement outcomes problematic [32,33,34].

**Figure 2:**
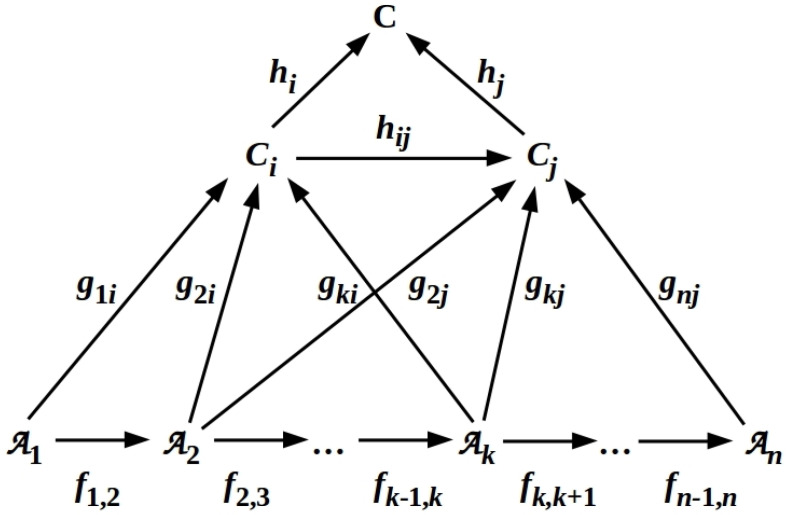
A co-cone diagram (CCD) is a commuting diagram depicting maps (infomorphisms) $f_{ij}$ between classifiers $A_{i}$ and $A_{j}$, maps $g_{kl}$ from the $A_{k}$ to one or more channels $C_{l}$ over a subset of the $A_{i}$, and maps $h_{l}$ from channels $C_{l}$ to the colimit C (*cf.* Equation 6.7 of Ref. [35]). Adapted from Ref. [10] Figure 3, CC-BY license.

Consider now a subset $\left\{M_{j}^{X}\right\}$ of the $M_{i}^{S}$ that act on some *m*-qubit subset $\left\{q_{j}\right\}$ of the *N* qubits composing B. The relationship between the *m* local *z* axes—the local reference frames for each of the *m* qubits—defines an overall reference frame for the sector *X* of B on which the $M_{j}^{X}$ act. Provided the internal Hamiltonian $H_{S}$ has sufficient degrees of freedom to implement this relationship, it constitutes a QRF [30,31]; in this case, we can write X=dom(Q), where *Q* is the implemented QRF. We have shown previously [11] that any QRF can be represented by a hierarchical structure, a cone–co-cone diagram (CCCD), of distributed information flow [35], the components of which are Barwise–Seligman [36] classifiers linked by maps (infomorphisms) that enforce logical consistency. A typical CCCD consists of a cone diagram (CD) and an attached complementary (i.e., all arrows are reversed) co-cone diagram (CCD) as illustrated in Figure 2 below. We note that any CCCD must be a commutative diagram, with the consequence that the local logic—and hence, the criterion of logical consistency—implemented by any subdiagram of a CCCD must also be commutative.

Just as any QRF—any subset of the $M_{i}^{S}$—alternately measures and prepares the states of some subset of qubits on B, any CCCD can be viewed as reading from and writing to an external system that effectively serves as a memory [8,35,37]. A CCCD is, therefore, a scale-free architectural blueprint for a massively parallel, distributed information-processing system, e.g., a variational autoencoder or a hierarchical Bayesian inference system as described in Refs. [8,37]. Each layer of a CCCD can, moreover, be viewed as both a metaprocessor over and an “internal” memory for the layers below it in the hierarchy. From this perspective, CCCDs provide a natural model of Global Workspace (GW) systems [37]. The colimit C in Figure 2, in particular, abstractly specifies such GW concepts as the *connective core* of Ref. [38], the *giant component* of Ref. [39], or other implementations of the original GW concept of a system that provides *access to consciousness* [40,41]; see Ref. [42] for further discussion in a biological context.

In the simplest case of a weighted, binary decision tree, the number of bits required to specify such a QRF, and hence the number of binary degrees of freedom required to implement it, scales as $m^{2}\log_{2}\left(m\right)$. In general, we can define the dimension dim(Q) of a QRF *Q* as $2^{M}$, where *M* is the number of binary degrees of freedom required to implement *Q*. Clearly, $\dim\left(Q\right)\gg \dim\left(X\right)=2^{m}$ whenever *m* is appreciably greater than one.

Just as *S* and *E* have free choice of local *z* axes for each of the $q_{i}$, they have free choice of QRFs, and hence free choice of how B is divided into sectors, with the limiting case of *S*’s QRF and sector choices determining *E*’s, or vice versa, again being entanglement [8]. Either *S* or *E* is free, moreover, to choose pairs $Q_{1}$ and $Q_{2}$ of QRFs that do not commute, i.e., such that $\left[Q_{1},Q_{2}\right]=Q_{1}Q_{2}-Q_{2}Q_{1}\neq 0$. Implementing noncommuting QRFs (equivalently, noncommuting diagrams having the form of CCCDs but for which the limit/colimit C is undefined) induces noncausal or “intrinsic” context dependence of both observations and actions implemented by an affected QRF [11,13]; see Refs. [13,37] for a comparison of this with other formalisms for describing contextuality, including contextuality-by-default [43] and the sheaf-theoretic formalism [44]. In particular, noncommutativity at the diagram level implies noncommutativity of the local logic of at least one subdiagram; see Ref. [45] for discussion of contextuality from this perspective. From an operational perspective, the I/O behavior of a QRF *Q* that is noncausally context-dependent appears to depend on a nonlocal (to *Q*) “hidden variable” that specifies a context [46,47,48]; from a theoretical perspective, noncommutative QRFs induce compartmentalization of *S* into bounded, separable components that can only communicate classically [49]. Noncommutativity, and, hence, the noncausal context dependence of QRFs can be induced by thermodynamic free energy limitations that force observations or actions using different QRFs to be performed sequentially [13]; hence, these effects can be expected to be ubiquitous in living systems.

## 4. No-Go Results for Generic Physical Interactions

We are now in a position to answer the question posed in the Introduction—to what extent can a generic physical system *S* observe, represent, and control its own internal processes?—by proving several “no-go” results that severely limit any physical system’s ability to represent its own internal states or processes. Employing the formal notion of a QRF allows us to state these limitations precisely. Because we are assuming only generic characteristics of physical systems and interactions, these limits apply very broadly, and can be challenged only by challenging fundamental—indeed axiomatic—assumptions of current physical theory. They are, therefore, comparable in both generality and strength to fundamental results from the theory of computation, such as the undecidability of the Halting Problem [50,51].

With the formalism defined in Section 2 and Section 3, we can state and prove the following.

**Theorem** **1.** *Let S be a finite system and Q be a QRF implemented by $H_{S}$. The following statements hold:*

1. *S cannot determine, by means of Q, either Q’s dimension dim(Q), Q’s associated sector dimension dim(dom(Q)), or Q’s complete I/O function.*
2. *S cannot determine, by means of Q, the dimension, associated sector dimension, or I/O function of any other QRF $Q^{\prime }$ implemented by S.*
3. *S cannot determine, by means of Q, the I/O function or dimension of any QRF $Q^{\prime }$ implemented by any other system $S^{\prime }$, regardless of the relation of S to $S^{\prime }$, from $S^{\prime }=S$ to $S^{\prime }=E$, inclusive.*
4. *Let $S=S_{i}S_{j}$, in which case $E_{i}=ES_{j}$. Then, $S_{i}$ cannot determine, by means of a QRF $Q_{i}$, the I/O function or dimension of any QRF $Q_{j}$ implemented by $S_{j}$.*

**Proof.**  We address each clause separately:

1. Any QRF *Q* accesses, by definition, $\log_{2}\left(\dim\left(\mathrm{dom}\left(Q\right)\right)\right)$ bits. As shown above, dim(Q)>dim(dom(Q)) for any *Q* of interest. No such QRF, therefore, has access to sufficient bits to count its own degrees of freedom, which it must do to specify dim(Q). Specifying dim(dom(Q)) requires specifying *Q*’s computational architecture, which requires specifying dim(Q). Specifying *Q*’s I/O behavior requires specifying dim(dom(Q)).
2. Unless $Q^{\prime }=Q$, in which case, see above, *Q* cannot access all of the bits composing $\mathrm{dom}(Q^{\prime })$ and hence cannot measure their states. Therefore, *Q* cannot determine the I/O function of $Q^{\prime }$. With no ability to count the bits in $\mathrm{dom}(Q^{\prime })$, *Q* cannot specify $\dim(\mathrm{dom}\left(Q^{\prime }\right))$. Specifying $\dim(Q^{\prime })$ requires specifying $\dim(\mathrm{dom}\left(Q^{\prime }\right))$.
3. Unless $S^{\prime }=S$, in which case, see above, *S* cannot measure the internal state $|S^{\prime }\rangle$, at least some components of which lie on the other side of the holographic boundary B, or determine the internal dynamics $H_{S^{\prime }}$. Hence, *S* can determine nothing about any $Q^{\prime }$ implemented by $S^{\prime }$.
4. As in this case $S_{i}\cap S_{j}=\emptyset$, the above case applies.

 □

Intuitively, Theorem 1 says that no physical system can determine its own observational capabilities (Clauses 1 and 2) or the observational capabilities of any other system (Clause 3). It also says explicitly that no component of a system *S* can determine the observational capabilities of any other component of *S* (Clause 4). Because Theorem 1 is stated in terms of QRFs, “observational capabilities” include the extraction of meaning from observational data. Theorem 1 therefore generalizes Quine’s classic result [52] that observers cannot deduce each other’s semantics by making it self-referential: it is also the case that no observer can deduce their own semantics. The opaqueness of their own minds to human observers has been emphasized by Chater [53] on psychological grounds; here, we obtain this same result via fundamental physics, in a form that applies to all physical systems.

For any classical system, Theorem 1 can be obtained from Theorem 2 of Moore [16], which shows that no finite sequence of finite-resolution I/O measurements can determine the function implemented by a classical Black Box. Crucially, the environment of any system surrounded by an MB is a Black Box for that system, with the MB serving as the I/O interface. Clauses # 1 and 2 above apply Moore’s theorem to I/O experiments performed by an observer on herself; clauses # 3 and 4 apply it in its originally intended setting of an observer interacting with an (at least partially) external system. The above proof can, therefore, be seen as simply extending Moore’s result to quantum systems.

Another classic result, Rice’s theorem [15], shows that the I/O function computed by an arbitrary system is undecidable by a Turing machine even if given the program implemented by the system. Hence, even providing *S* with a program for some *Q* will not, in general, allow *S* to determine the I/O behavior of *Q*.

Three corollaries follow immediately from Theorem 1:

**Corollary** **1.** *Let $S=S_{i}S_{j}$. $S_{i}$ cannot act on $S_{j}$ to specifically induce a map $Q_{j}\mapsto Q_{j}^{\prime }$ from a QRF $Q_{j}$ implemented by $S_{j}$ to a $Q_{j}^{\prime }$ determined by $S_{i}$.*

**Proof.**  From Theorem 1, $S_{i}$ cannot determine that $S_{j}$ implements either $Q_{j}$ or $Q_{j}^{\prime }$, so it cannot act specifically to induce a map from one to the other. □

**Corollary** **2.** *$S_{i}$ cannot detect context shifts that induce maps $Q_{j}\mapsto Q_{j}^{\prime }$ in $S_{j}$.*

**Proof.**  From Theorem 1, $S_{i}$ cannot determine that $S_{j}$ implements either $Q_{j}$ or $Q_{j}^{\prime }$, so it cannot detect context shifts that induce a map from one to the other. □

Corollary 1 shows that a component $S_{i}$ of a system—e.g., a metaprocessor or “executive” component—cannot act specifically to control the behavior of another component $S_{j}$. Components of a system can act on each other but cannot deterministically control each other’s behavior. Corollary 2 shows that a component $S_{i}$ cannot determine what causes changes in the behavior of another component $S_{j}$. Corollary 2 allows $S_{i}$ to detect contextuality in the statistics of $S_{j}$’s behavior but restricts $S_{i}$ from determining what a detectably different context is for $S_{j}$. A system $S_{i}$ can, in other words, determine by observation that another system $S_{j}$ is acting with non-codeployable QRFs, but because it cannot determine what those QRFs are, it is unable to determine how $S_{j}$ distinguishes different contexts. It cannot, in particular, determine at what level in $S_{j}$’s processor hierarchy—at what level in $S_{j}$’s GW—operators become non-codeployable. Hence, $S_{i}$ cannot fully reverse engineer $S_{j}$’s attention system “from the outside”, though it can determine that $S_{j}$ is employing attentional shifts.

**Corollary** **3.** *The models implemented by physical systems are incomplete in the sense that there are inputs that can be received but not predicted, and adding more or different QRFs or hierarchical (i.e., meta) layers cannot make them complete.*

**Proof.**  Clause 2 of Theorem 1 restricts any system *S* from determining the QRFs implemented by its environment *E*, and therefore from modeling them with complete accuracy from its observations. It similarly prevents *E* specifically acting on *S* to adjust *S*’s QRFs toward a model of *E*. The FEP acting on the *S*-*E* system will drive them asymptotically toward zero prediction error and hence shared QRFs; however, this asymptotic state is entangled [8,54], rendering the *S*-*E* distinction physically meaningless. □

Reading “received” as “true” and ”predicted” as “provable”, Corollary 3 can be seen as an analog, in the current setting, of Gödel’s celebrated first incompleteness theorem [55]. Gödel showed that any finite system of axioms is insufficient to prove every result in mathematics, or otherwise said that in any logically consistent axiomatic system with sufficient richness to express arithmetic, there will always be both truths and untruths that can neither be proved nor disproved within the axioms of that system. Like Gödel’s theorem, Corollary 3 turns on the notion of finite construction (of proofs or predictions) and on the contradictory (physically meaningless) nature of perfectly self-referential statements.

Theorem 1 and these three corollaries do not imply that systems cannot have models of themselves, or that metaprocessors within larger systems cannot use models of object-level components when acting on such components to influence their behavior. Theorem 1 and its corollaries rule out both the inductive construction of such models from observational data and empirical testing of such models using observational data. We are left with the conclusion that “self-models” at either the object- or the meta-level can only be heuristic, can only be learned under environmental supervision, and cannot converge to completeness, and hence perfect predictive accuracy, without destroying the identity—the distinctness from its environment—of the system that implements them.

## 5. Examples

The above results show that “self” models are subject to the same restrictions as “other” models, specifically, models of the environment [13]. Indeed, they show that “self” models are “other” models—they are models of an object component $S_{j}$ that are implemented by a meta component $S_{i}$ of some composite system *S*. To distinguish between a system’s self-model and a model constructed by an external observer, and to examine the heuristics used in either kind of model, it is useful to consider some specific examples.

### 5.1. Example: Hawking’s Speculation

Hawking in his Dirac Centennial lecture [56], on reviewing possible amalgamations of string theory, quantum gravity, and M-theory, lends doubt to the possibility of ever achieving a complete theory of the universe in terms of a finite number of statements. This is likened to Gödel’s theorem [55] mentioned above, in that it associates completeness with self-contradiction. Hawking’s speculation can be based on the observation that any physical theory is self-referencing, and can be expected to be inconsistent or incomplete, with present-day physical theories deemed by Hawking to be both (supporting evidence is discussed in [57,58,59]). Such speculation can in part be traced back to Wheeler’s earlier contention [60] that any quantum state is self-observable, thus leading some to suggest that the paradoxical nature of quantum theory is due to one of self-reference, and to determine whatever is the underlying cause for incompleteness/undecidability (e.g., [45]). Inspired by the original work of von Neumann [61], these questions have been approached from the theories of noncommutative logic and algebras (e.g., Refs. [45,62,63]). In this respect, we note that a noncommuting diagram with the form of a CCCD exhibits a noncommutative system of logic infomorphisms as based on the (local) logics of Ref. [36] as recalled and reviewed in Ref. [37]. In particular, the general nature of Corollary 3 here provides strong evidence for Hawking’s claim, while also posing startling consequences for Wheeler’s contention [64] that physics is fundamentally about information exchange, as well as the claim that physics is about language, professed by Grinbaum [65].

### 5.2. Example: Heisenberg Uncertainty

In Ref. [66], it is shown that Heisenberg’s Uncertainty Principle (HUP) implies algorithmic randomness [67,68], which in turn implies Chaitin’s notion of informational incompleteness [69], the latter being a form of incompleteness due to Gödel [55,70]. Relevant here is how the steps leading to the “no-go” results of Section 4 implicitly involve an algorithmic complexity as generated by qubit strings along B. Such complexity is already implicit in the Frame and Halting problems [71,72] demonstrated to be undecidable as, indeed, is the Quantum Frame Problem [13]. The HUP, as a principle of indeterminacy, has also been shown to be a form of quantum contextuality in Ref. [73]. These results entice further exploration of the prospectively deep connections between indeterminacy, incompleteness/undecidability and contextuality, and indeed between quantum theory and metamathematics, to be pursued in view of Theorem 1 and its corollaries at a later date.

### 5.3. Example: Supervised Learning

In an artificial neural network (ANN) undergoing supervised learning via an algorithm such as error back-propagation, inputs arrive at alternate times from one of two sources, the task environment or the supervisor. These inputs are processed by two, non-codeployable QRFs: inputs from the task environment are processed by the ANN units, with the interprocessor connection weights fixed, while supervisory inputs are processed by the connections, to update their assigned weights, without affecting the states of the units. Switching between these input regimes is controlled by a metaprocessor implemented either in hardware or, in simulated ANNs, in software. Following training, both the metaprocessor and the weight-updating QRF are turned off so that the ANN processes inputs from the task environment only. This being the case, a well-programmed ANN nevertheless strives to mimic some “optimal” computational task in attaining to the “Good Regulator” Theorem of Ref. [74].

Let us call the metaprocessor $S_{2}$ and the “object-level” ANN $S_{1}$. The environment of $S_{1}$ comprises the task environment, the supervisor, and $S_{2}$; the input from $S_{2}$ is, without loss of generality, the value of a control bit that selects one of the two object-level QRFs. The activation states of any one of $S_{1}$’s units encodes, and therefore represents, the input from the task environment as processed by all upstream units; this representation is available only to the downstream units, and only for further processing. The connection weights encode, and therefore represent, the training inputs; each connection’s representation is available to it alone, and only for execution. The value of the control bit encodes an input from $S_{2}$, and is available to the QRF switch only for execution. As required by Theorem 1, no component of $S_{1}$ has access to either of the QRFs that $S_{1}$ implements, to $S_{1}$’s overall architecture (i.e., the number of units or their connection weights), or to the function that $S_{1}$ computes at any stage of training.

The restrictions imposed by Theorem 1 apply equally to $S_{2}$, which has no access to any of the above information about $S_{1}$, and no access to its own state-switching algorithm. Indeed, $S_{2}$ could be implemented simply by a flip/flop, or by an external switch operated by a user. While it would be straightforward to add a reporting component to $S_{2}$ that announced when the ANN was being switched from processing to training mode and vice versa, this would not affect the representations available to the $S_{2}$. Routing this information to $S_{1}$ would similarly have no effect on $S_{1}$, as it would always have a constant value in either $S_{1}$’s processing or its training mode.

Theorem 1 restricts any ANN from representing to itself whether it is in training or processing mode, though its outputs can make this difference evident to an external observer. Replacing the notion of representation with a first-person notion of a phenomenological “in the world—lived experience” [75] does not change this conclusion; $S_{1}$ experiences input streams, not its own processing, while $S_{2}$ only experiences a one-bit state change. Making the “supervisor” a component of the system, as in a Generative Adversarial Network (GAN), also does not change the conclusion; neither the supervisor component nor the combined system has access to either the overall architecture or any of the computed functions. These restrictions have clear relevance for the explanation problem [76,77] that besets ANN designers and users, particularly designers and users of multi-layer deep learning systems. Such systems may report explanations of their computational behavior, but cannot, by Theorem 1, have full access to either the computations being explained or the computations being used to explain them. In this respect, self-explaining ANNs are similar to humans, who employ heuristics and confabulation to explain their behavior [53] as discussed further below.

### 5.4. Example: Reinforcement Learning

Gene regulatory networks (GRNs) can be trained toward novel attractors, and hence exhibit memory capabilities [78,79]. More generally, stochastic networks, including liquid-state physical systems, can be trained toward novel attractors, and hence exhibit memory capabilities [80]. In any such system, we can draw a boundary around some set $S_{1}$ of nodes or elements, and ask what $S_{1}$ can represent about both itself and its environment, i.e., the external environment plus the remaining system component $S_{2}$. Theorem 1 places restrictions on what $S_{1}$ can represent analogous to those above. In this case, as in the above, $S_{1}$ “experiences” information flow across its boundary, but does not experience either its own internal processes or those of $E⊕S_{2}$.

### 5.5. Example: Self-Editing Systems

The introduction of LISP as a programming language in the early 1960s [81] made self-editing systems feasible targets for implementation in software. Self-editing, generally realized as the editing of an object-level component $S_{1}$ by a meta-level component $S_{2}$, is foundational for autonomous learning. Architectures as diverse as CLARION [82], LIDA [83], and MACSi [84] that support autonomous learning provide examples; see Ref. [85] for a comparison of multiple such architectures. Each module in such an architecture represents only what its interface—effectively, its MB—with the rest of the system allows it to represent. In accord with Theorem 1, meta-level modules cannot fully determine, either in advance or *post hoc*, the effects of a software change on the behavior of a targeted object-level module in its own environment. Such systems face, effectively, the same explanation problem as that faced by human engineers supervising training of an ANN.

All organisms are self-editing systems that engage in autonomous learning. Gene expression, for example, can be seen as self-modification of cellular biochemical state, as can developmental bioelectrical signaling that alters the state of an electrical circuit and hence what activity/computations can be performed next [86,87]. The GRNs that control gene expression have access only to highly coarse-grained representations of the cellular states they are modifying—those encoded by the second messenger systems with which they directly interact—and cannot, in particular, distinguish state changes due to external inputs from internally generated state changes. Hence, GRNs, like human engineers or evolution itself [88], are tinkerers, not fully informed planners that can determine a desired outcome in advance.

### 5.6. Example: Intrusion Detection

Immune systems, from microbial restriction enzymes to mammalian B, T, and NK cells, are often described informally as distinguishing “self” from “other” and eliminating the latter. At the component level of description, however, such systems are only engaging in molecular recognition; the source of the recognized ligand is irrelevant, as auto-immune diseases reveal. Intrusion-detection software works in a similar way, flagging or deleting anything meeting some specification, regardless of its source. Such systems are, therefore, representations of “self” only in a negative sense: anything not recognized as “other” is treated as “self”. A representation of this kind does not bound the self, and supports no inferences about the self’s behavior; hence it presents no conflict with Theorem 1.

### 5.7. Example: The Human Narrative Self

Evidence from functional neuroscience increasingly supports the hypothesis that the human narrative self—what people typically describe when asked to describe themselves—is a *post hoc* construct implemented largely by the theory-of-mind (ToM) components of the default mode network [89,90,91,92,93]. This representation integrates current interoceptive, affective, and perceptual data with autobiographical memories that are now widely acknowledged to be at least partially confabulatory [94,95] as further discussed in Section 5.8 below. It depends on brainstem inputs not only for sufficient arousal but also for the non-representational “feeling of being alive” [96]. It is not maintained continuously but is severely attenuated if not absent during activities that require externally focused attention, particularly in flow states [97] but more generally during activities with some degree of automaticity, including everyday activities such as social interaction and language use [98,99]. Attenuation of the narrative self representation is a typical goal of meditation practices [100,101,102], and a typical effect of psychedelics [103,104]; it contributes to the therapeutic effect in both [105].

In the context of the FEP, the construction of the narrative self is an object-level process that can be activated or attenuated by a meta-level process that allocates attention, or in Bayesian terms, modulates precision assignments to priors [106,107]. Reducing the narrative self to a representation constructed by an object-level process removes it from the “driver’s seat” of cognition that it describes itself as occupying—a position it has enjoyed in theories of cognition at least since Descartes, despite challenges from Freud and others—and makes it merely one of several passengers [53]. The primary target of active inference as “self-evidencing” [5] is, therefore, not the narrative self, which needs no evidence, but rather the environment, which observes the self-evidencing system as a whole. This demotion of the narrative self to a *post hoc*, coarse-grained representation of whole-system behavior, including some attended-to sample of cognition, is clearly consistent with the restrictions placed on self-representation by Theorem 1 and its corollaries. It does not, however, alter the utility of the narrative self as an apparent locus of overall behavioral control, particularly its utility to external observers equipped with their own ToM systems. As external observers, we can marvel at the “self-control” exhibited by, for example, athletes or musicians, even when they are operating in pure flow states and have no *post hoc* reportable experiences of their narrative selves. We can, indeed, marvel via our narrative selves at our own performance while in such states, provided we engage in such self-reflection only after such a performance has been completed. Attempting to do so in real time disrupts the flow, rendering the performance clunky and amateurish [97,106], and in critical situations, possibly fatal.

### 5.8. Example: Cognitive Biases and Confabulation

The outline of the example in Section 5.7 suggests that psychological “effects” that reveal incomplete or faulty self-knowledge are to be expected. These include cognitive biases that typically over-estimate knowledge or the reliability of memory, as well as various forms of motivated self-deception [108,109,110,111,112], a likely cause of how introspection/contemplation can disrupt cognitive processes. An original approach to this question was taken up by Nisbett and Wilson in Ref. [113], who studied individual self-knowledge by noting that when subjects were asked to explain their behavior in certain situations, they revealed a *dependence on shared theories* concerning the causes of their behavior, rather than the *actual causes* of the latter. Further studies in this direction proposed that thinking can undermine the relationship between an individual’s attitudes and behavior (reviewed in Ref. [114]), and to a broadly accepted suggestion that when explaining some attitude, responses are often, to some degree, confabulated. In recent years, confabulation in relationship to self-knowledge has received growing attention in psychological, philosophical and neuroscience studies, for instance, in distinguishing confabulation types [115], e.g., as a memory distortion (reviewed in Ref. [116]), and various nuances of the meaning of the term (e.g., Ref. [117]: a motivation by the desire to have fulfilled a rational obligation to explain attitudes by reference to motivating reasons; Ref. [116]: a distortion of a specific form of consciousness allowing individuals to locate objects and events according to their subjective temporality). From a clinical perspective, frequent confabulation was observed in young autistic subjects in Ref. [118], who suggested the cause as due to memory impairment and an executive control condition, more so than the subjects’ actual milieu.

## 6. Discussion

One fundamental aspect highlighted by these results is the boundary of the apparent self—called the “Self” in Ref. [119]—what the narrative self describes in the case of humans. It is the Self that systems use to distinguish themselves from the outside world. This is especially critical for biological beings—including both conventional cognition (brain-based operation in the 3D world) and the kinds of unconventional diverse intelligence exhibited by non-neural cells, tissues, and organs operating in physiological, transcriptional, and anatomical problem spaces [120]. Establishing models of the Self and its boundary is important for the efficiency of life (e.g., estimating what effectors one has, and which aspects of the world can be “directly” controlled and which cannot), and for causal intrusion detection needed for resistance to parasites and cheaters (e.g., “did I do that, or is some other agent hacking me?”). It is also essential for the powerful ability to coarse-grain events in the world to tell (whether consciously or implicitly) agential stories about oneself and others, which allow a very compressed and effective interface for control and cooperation. As noted above, these are uses to which the narrative self is put by humans.

Recent work formalized some of these ideas, developing the concept of the Cognitive Light Cone (CLC), which represents the spatial and temporal limits on the size of goals that a given agent can represent and pursue [119]. The Technological Approach to Mind Everywhere (TAME) framework [3] focuses on how individual competent subunits, such as cells, can join into collectives (networks), which can pursue much larger goals in novel problem spaces, thus increasing their CLCs. According to this framework, Selves are some observer’s (including the system’s own) model of a triad consisting of a space within which the system operates, a specific CLC, and a set of competencies that the system is able to deploy to navigate that space. This fundamentally emphasizes the fact that the extent of Selves is not obvious (e.g., at the skin of an organism or a cell’s plasma membrane) but is the subject of an active construction and modeling task that it or some external observer must perform. This naturally raises the issue of the limits on the efficacy of that self- and other-identifying process, some of which have been made explicit here. Another consequence of the TAME substrate-independent account of agency [42] is that Theorem 1 implies that the environment is a better judge of a system’s CLC than the system itself is Ref. [121]. This impacts both biological applications of the autopoietic construction of the Self–world boundary (for evolutionary developmental biology, regenerative medicine, and psychiatry [122,123,124]), and the social/personal impact of increasing understanding of what we really are [125,126].

## 7. Conclusions

We have shown here that there are principled limitations on self-representation that derive from fundamental physical considerations and therefore apply to all physical systems. These limitations follow from the fact that the boundaries separating—and thereby distinguishing—systems from their environments function as MBs. They therefore apply, in particular, to all systems characterized by the FEP, even to systems that do not have obvious or time-stable boundaries in ordinary 3D space.

Our results show that while metaprocessors that generate self explanations may contribute to resolving the explanation problem for ANNs, they cannot solve it. Indeed, we can expect ANNs—and multi-layer systems in particular—to confabulate or “hallucinate” self-explanations just as humans do. Our results also both confirm and provide a fundamental physics grounding for Brook’s claim that fully centralized control systems cannot work [127], though we note that this grounding has nothing to do with an absence of representations. More broadly, they show that cognition—in particular, observation, representation, and control—must be considered to be both embodied (i.e., physically implemented) and enactive (i.e., include action on the environment as an information acquisition strategy). These are, therefore, fundamental physical requirements for cognition, not philosophical options; indeed, they are requirements for any active inference system [9].

The role of fundamental no-go results in science is to show that blind alleys really are blind. In physics, the fundamental no-go theorems of Bell [46,128] and Kochen-Specker [47] gave birth to quantum information theory. Gödel’s theorem substantially motivated the founding of computer science. Our results, effectively, bring neuroscience closer to physics, suggesting that in the long term, one has to square up to the possibility of incompleteness/undecidability arising in a field that has been traditionally deterministic, or at least classically stochastic. From the cognitive perspective, a general explanation can be given in terms of the “no-go” results (particularly Corollary 2) that suggest individuals to be Black Boxes to themselves, at least partially dissolving introspective self-knowledge and replacing it with self-model heuristics and confabulation. This can be expected to have consequences for recent models including the “inner screen” [107], “interface” [129], and “beast machine” [93] approaches, among others. Moreover, it is a matter that applies to all cognitive systems, from the basal level upwards [42], whose cellular/nervous systems are in constant contention with an environment that is all-too-uncertain, if not patently hostile.

We recognize that results such as ours have implications for ethical and legal theories of responsibility and intent [112,130], for political neuroscience [131], and even for fundamental questions of personal identity and the “meaning of life” [132]; however, consideration of these issues is beyond the present scope. We do hope that our current results will help to motivate a final rejection of the homuncular idea of centralized controllers in favor of a fuller understanding of the distributed nature of observation, representation, and control in both natural and artificial systems. From a more humanist perspective, we hope that it encourages a greater appreciation of uncertainty and embodiment as essential components of intelligence and awareness, and a fuller understanding of what it means to be an active embodied mind.

## Data Availability

Data are contained within the article.

## Abbreviations

The following abbreviations are used in this manuscript:

ANN	Artificial Neural Network
CCCD	Cone–Co-Cone Diagram
CLARION	Connectivist Learning with Adaptive Rule Induction On-line
CLC	Cognitive Light Cone
DL	Deep Learning
FEP	Free Energy Principle
GAN	Generative Adversarial Network
GRN	Gene Regulatory Network
GW	Global Workspace
HP	Holographic Principle
HUP	Heisenberg’s Uncertainty Principle
I/O	Input/Output
LIDA	Learning Intelligent Distribution Agent
LISP	List Processing
MACSi	Motor Adaptive and Cognitive Scaffolding for iCub
MB	Markov Blanket
QRF	Quantum Reference Frame
TAME	Technological Approach to Mind Everywhere
ToM	Theory of Mind
